# Examination of duodenal and colonic microbiome changes in mouse models of acute and chronic pancreatitis

**DOI:** 10.1038/s41598-024-75564-1

**Published:** 2024-10-21

**Authors:** Rabea Lange, Juliane Glaubitz, Fabian Frost, Andreas Geisz, Ali A. Aghdassi, F. Ulrich Weiss, Matthias Sendler

**Affiliations:** 1 Department of Medicine A, University Medicine Greifswald, Fleischmannstr. 41, 17475 Greifswald, Germany; 2 Department of Surgery, Boston University Chobanian and Avedisian School of Medicine, Boston, MA USA

**Keywords:** Acute pancreatitis, Chronic pancreatitis, Microbiome, Inflammation, Gastroenterology, Pancreatitis

## Abstract

The exocrine pancreas is the main source of digestive enzymes which are released from secretory vesicles of acinar cells into the small intestine. Enzymes, including amylases, proteases and lipases, degrade the ingested food and thus determine the nutritional substrate for the gut microbiota. Acute (AP) and chronic pancreatitis (CP) are associated with a transitional or progressive exocrine pancreatic dysfunction, we analysed in the present study how an experimental induction of pancreatitis in mouse models affects the colonic and duodenal microbiome composition. Evaluation by 16 S rRNA gene sequencing revealed specific microbiome changes in colonic as well as in duodenal samples in different models of AP and CP. Mild acute pancreatitis, which is associated with a transient impairment of pancreatic secretion showed only minor changes in microbial composition, comparable to the ones seen in progressive dysfunctional mouse models of CP. The strongest changes were observed in a mouse model of severe AP, which suggest a direct effect of the immune response on gut microbiome in addition to a pancreatic dysfunction. Our data indicate that highly dysbiotic microbiome changes during pancreatitis are more associated with the inflammatory reaction than with a disturbed pancreatic secretion.

**Supplementary Information:**

The online version contains supplementary material available at 10.1038/s41598-024-75564-1.

## Introduction

Acute pancreatitis (AP) is one of the most common non-malignant diseases of the gastrointestinal tract leading to hospital admission. While about 80% of patients have a self-limiting course of disease, about 20% of patients develop severe necrotizing pancreatitis, which is associated with life-threatening complications such as multiple organ failure or infection of pancreatic necrosis by commensal intestinal bacteria^1,2^. Furthermore, about 17–35% of AP patients experience recurrent pancreatitis and/or develop a chronic form of the disease^3–5^, which is associated with severe limitations in quality of life. Disease progression leads to an increasing loss of pancreatic function as exocrine and endocrine tissue becomes replaced by fibrotic tissue, which may ultimately leads to complete pancreatic insufficiency^6^.

Chronic pancreatitis (CP) as well as acute episodes of CP impair pancreatic function which associates with changes in the gut microbiome composition^7^. Digestive enzyme secretions such as amylase, proteases and lipases from the secretory compartment of pancreatic acinar cells influence the composition of the nutritional substrate for the gut bacteria^8^. Population-based studies have shown that a reduced stool elastase content, which is a marker for pancreatic dysfunction, is one of the strongest factors that cause gut microbiome changes, more than other factors like age, gender or BMI^7^. In chronic pancreatitis patients significant changes of the gut microbiome could be detected, as facultative pathogenic bacteria were significantly more abundant^9^. Beside population-based studies, experimental animal studies were performed to investigate disease related changes of the gut microbiome. These studies have shown that the intestinal microbiome has a decisive influence on the disease course and severity of acute pancreatitis^10–12^. During AP a secretory blockade of acinar cells results in reduced release of digestive enzymes into the duodenum. In our previous study, we could show that in the course of severe AP in mice the gut microbiome changes significantly and facultative pathogens increase in abundance especially in the duodenum^10^. These findings raised the question if the reported gut microbiome alterations are merely caused by a disturbed pancreatic enzyme secretion, or whether other factors are involved that influence the microbiome composition. In addition to the altered digestive enzyme secretion, acute episodes of pancreatitis trigger a strong systemic immune response^10,13,14^. The aim of this study was to investigate the causative factors for gut microbiome composition changes in mouse models of acute and chronic pancreatitis with different degrees of severity. Two models of CP with different degrees of impaired pancreatic secretion^17,18^ were compared with two AP models with significant different inflammatory reactions^10^. Microbial changes in colon and duodenal samples from these mouse models of pancreatitis were analysed by 16 S rRNA gene sequencing. Recent studies are also investigating the diagnostic and prognostic value of the microbiome composition^1516^. Our study further analysed the dysbiosis associated with distinct models of AP and CP and if the microbial changes have prognostic or diagnostic potential.

Our data suggest that both pancreatic function and acute inflammation have a severity-dependent impact on the composition of the gut microbiome. While mild forms of acute and chronic pancreatitis showed only minor changes of the microbiome, severe form of the disease results in a significant dysbiosis, especially in the duodenum.

## Results

### The gut microbiome in two mouse models of chronic pancreatitis

**Fig. 1:**
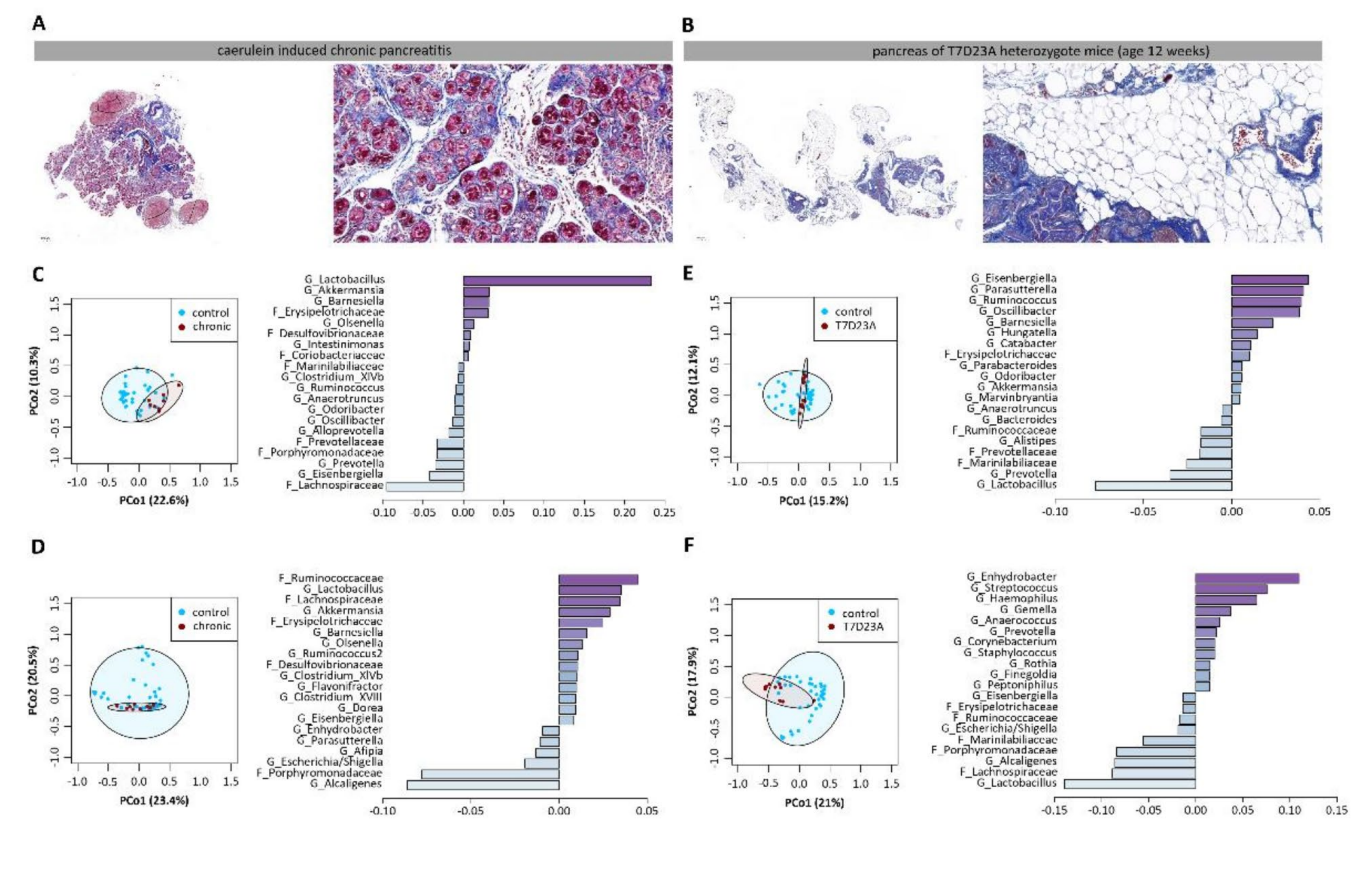
Gut microbiome analysis in two mouse models of chronic pancreatitis. **A**, **B** Azan blue staining of pancreatic tissue of caerulein induced CP and T7D23A heterozygous mice. Faecal samples from colon and duodenum were collected from C57Bl/6-J mice after CP induction (*n* = 8), from T7D23A mice (*n* = 11) and from control animals (*n* = 39). Isolated DNA was analysed by 16 S rRNA gene sequencing. **C** Principal coordinate analysis illustrates the changes of gut microbiome between controls and CP mice in colonic samples (R^2^ = 14.14%, *p* ≤ 0.001), a bar graph illustrates the 20 taxa with the highest fold change in the relative abundance of the CP group in relation to controls. **D** PCoA (R^2^ = 7.00%, *p* = 0.01) and bar graph illustrate the changes in the duodenal microbiome. **E**, **F** PCoA illustrate the microbiome in colon (R^2^ = 7.17%, *p* ≤ 0.001) and duodenum of T7D24A mice compared to controls (R^2^ = 15.21%, *p* ≤ 0.001). Bar graphs showed the most affected taxa. Significant changes were tested by PERMANOVA.

We investigated the gut microbiome in two mouse models of CP. In the first model, we induced CP in C57Bl/6-J mice by repetitive i.p. injections of caerulein over a period of 4 weeks as previously described^18^. Azan blue staining illustrates the degree of fibrosis, as well as the remaining residual pancreatic acinar cells (Fig. 1A). As a second disease model, we chose a genetically modified mouse model where the animals carried a mutation in the activation peptide of the murine T7 trypsinogen gene (T7D23A) which leads to an autoactivation of trypsinogen^17^. The animals develop episodes of acute pancreatitis at the age of 3–5 weeks resulting in a complete loss of function of the exocrine pancreas after 8–12 weeks. Their exocrine tissue gets completely replaced by fatty tissue (Fig. 1B). Colonic as well as duodenal samples were collected from CP and control mice for 16 S rRNA gene sequencing. Principal coordinate analysis (PCoA) showed a spatial separation between control mice and mice with caerulein induced CP in colon samples as confirmed by permutational analysis of variance (R^2^ = 14.14%, *p* ≤ 0.001) (Fig. 1C) and duodenal samples (R^2^ = 7.00%, *p* = 0.01) (Fig. 1D). Bar graphs showed the most significantly affected 20 taxa compared to controls. Colonic as well as duodenal samples of T7D23A heterozygote mice were also collected and analysed by 16s rRNA gene sequencing. PCoA followed by permutational analysis of variance illustrate significant differences in microbiota composition between control mice and T7D23A heterozygote mice in colon (R^2^ = 7.17%, *p* ≤ 0.001) (Fig. 1E) as well as duodenal samples (R^2^ = 15.21%, *p* ≤ 0.001) (Fig. 1F). Bar graphs show the most significantly affected 20 taxa in T7D23A heterozygote mice compared to controls. In both the experimentally induced model of CP and the genetic model of T7D23A mice, we observed significant CP-dependent changes in the composition of the gut microbiome in colon as well as in duodenum samples.

### The gut microbiome in two mouse models of acute pancreatitis

**Fig. 2:**
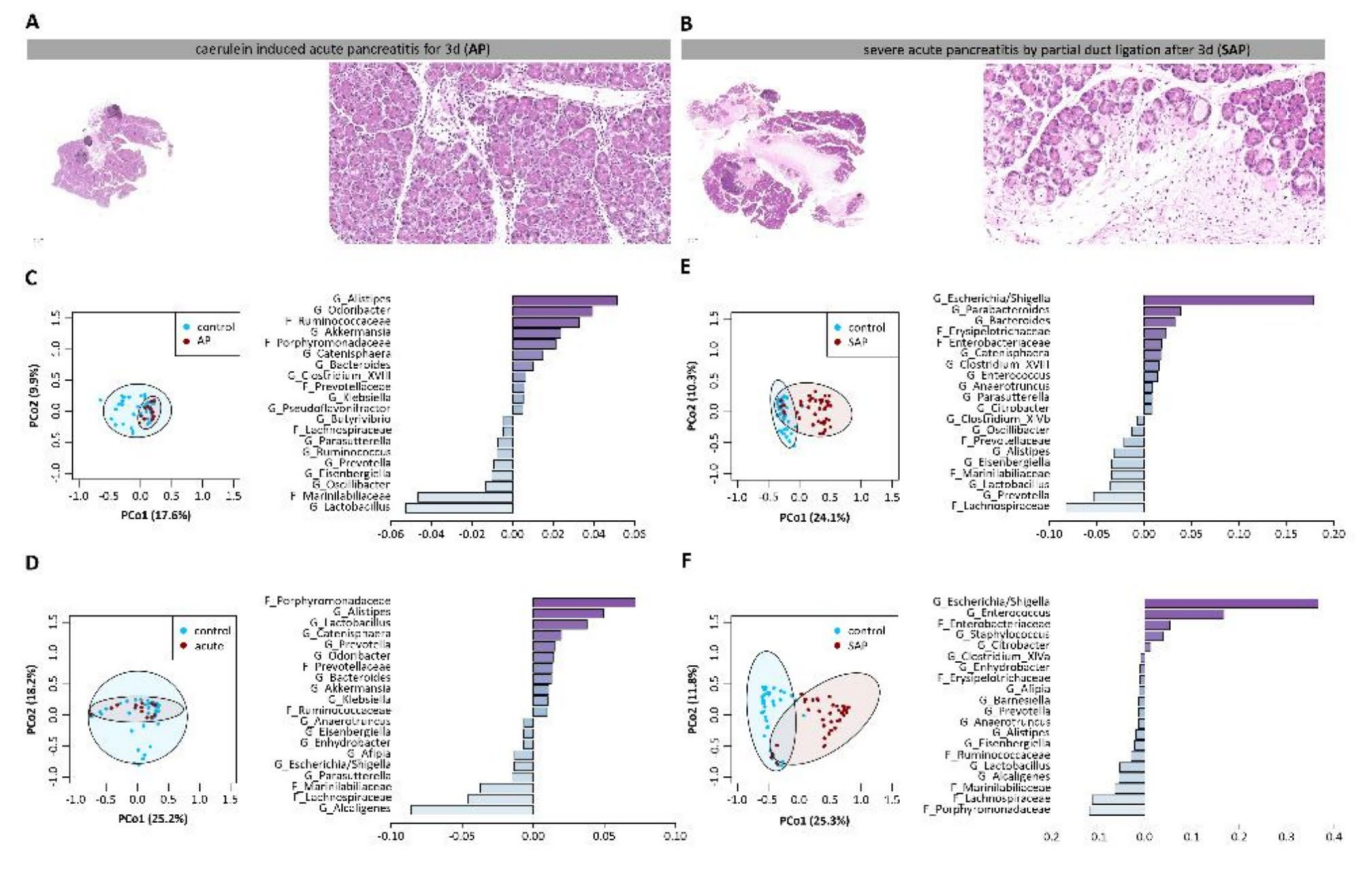
Gut microbiome analysis in two mouse models of acute pancreatitis. **A**, **B** H&E staining of pancreatic tissue after AP induction by caerulein and severe AP (SAP) induction by pancreatic duct ligation. Faecal samples from colon and duodenum were collected from C57Bl/6-J mice after AP induction (*n* = 15), from SAP mice (*n* = 44) and from control animals (*n* = 39). Isolated DNA was analysed by 16 S rRNA gene sequencing. **C**,** D** Principal coordinate analysis illustrates gut microbiome changes between controls and AP mice in colonic samples (R^2^ = 8.88%, *p* ≤ 0.001) (**C**) and duodenum samples (R^2^ = 4.77%, *p* = 0.029) (**D**). Bar graphs show the 20 most up-or downregulated taxa. **E**, **F** PCoA illustrates the microbiome changes of SAP mice compared to controls in colon (R^2^ = 23.78%, *p* ≤ 0.001) and duodenum (R^2^ = 31.52%, *p* ≤ 0.001), bar graphs show the 20 most affected taxa. Significant changes were tested by PERMANOVA.

Next, we investigated the gut microbiome in two mouse models of AP. Both models are well established and described in detail in our previous work^10^. A mild AP was induced using 8 hourly i.p. injections of caerulein for three consecutive days in C57Bl/6-J mice. H&E histology showed marked infiltration of immune cells into the damaged organ, as well as single necrotic acinar cells (Fig. 2A). In a second model, we induced severe necrotizing pancreatitis by partial ligation of the pancreatic duct. In this model, the pancreatic duct is ligated using a surgical suture, followed by a single caerulein administration two days after the procedure. Comparable to the mild AP model, we analysed the animals 72 h after ligation. H&E histology showed extensive necrosis in the ligated part of the pancreas and infiltration of immune cells (Fig. 2B). This model resembles severe acute pancreatitis (SAP). The intestinal microbiome was examined in colon as well as in duodenum. PCoA, followed by permutational analysis of variance, showed significant differences in microbiota composition between control mice and mice with caerulein induced mild AP in colon (R^2^ = 8.88%, *p* ≤ 0.001) (Fig. 2C) and duodenal samples (R^2^ = 4.77%, *p* = 0.029) (Fig. 2D). PCoA of SAP mice showed also significant differences in microbiota composition between controls and SAP mice in colon (R^2^ = 23.78%, *p* ≤ 0.001) (Fig. 2E) as well as duodenal samples (R^2^ = 31.52%, *p* ≤ 0.001) (Fig. 2F). Bar graphs showed the most significantly impacted 20 taxa compared to controls. In both AP models we observed significant changes of the microbiome in colon as well as in duodenum samples.

### Microbiome composition changes in colon and duodenal samples

**Fig. 3:**
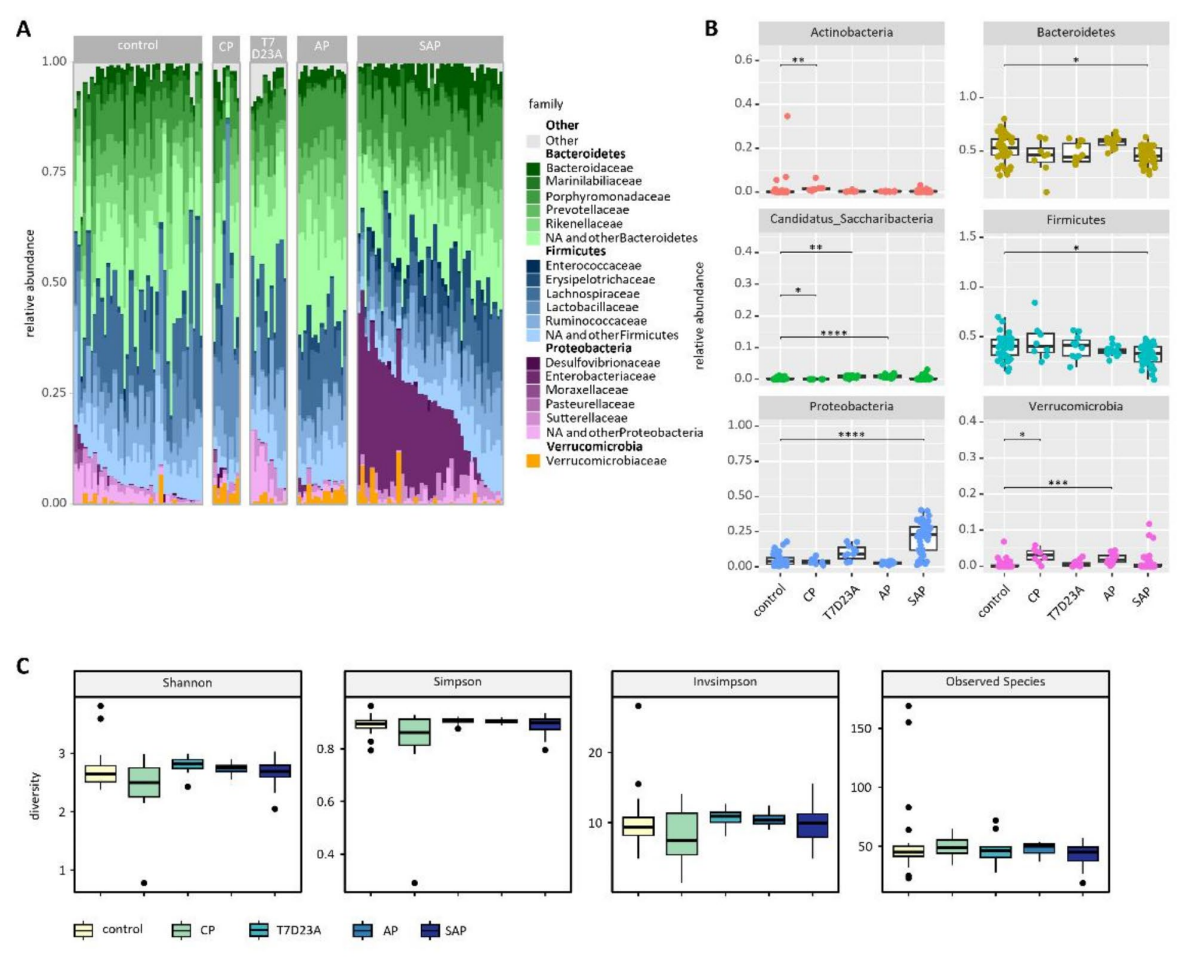
Changes of the colonic microbiome in experimental pancreatitis models. (**A**) A stacked bar graph shows the most abundant taxa of the four most prominent phyla. (**B**) Box plots illustrate the relative abundance of the six most prominent phyla in general. (**C**) Box plots show the diversity index (Shannon, Simpson and Invsimpson) and the richness (observed species) in colon samples. Significant changes were tested by Wilcoxon-test followed by correction for multiple testing.

We also analysed for differences in the microbiome composition between acute and chronic pancreatitis. A stacked Bar Plot illustrates the top 4 phyla with the 5 most represented families in colon samples of all models (Fig. 3A), and box plots show the relative abundance of the 6 most common phyla between the different murine models of acute and chronic pancreatitis (Fig. 3B). Especially in SAP animals a significant redistribution from Bacteroidetes and Firmicutes to Proteobacteria was observed. However, the general diversity or species richness in the colon samples was not altered in acute or chronic pancreatitis (Fig. 3C).

**Fig. 4:**
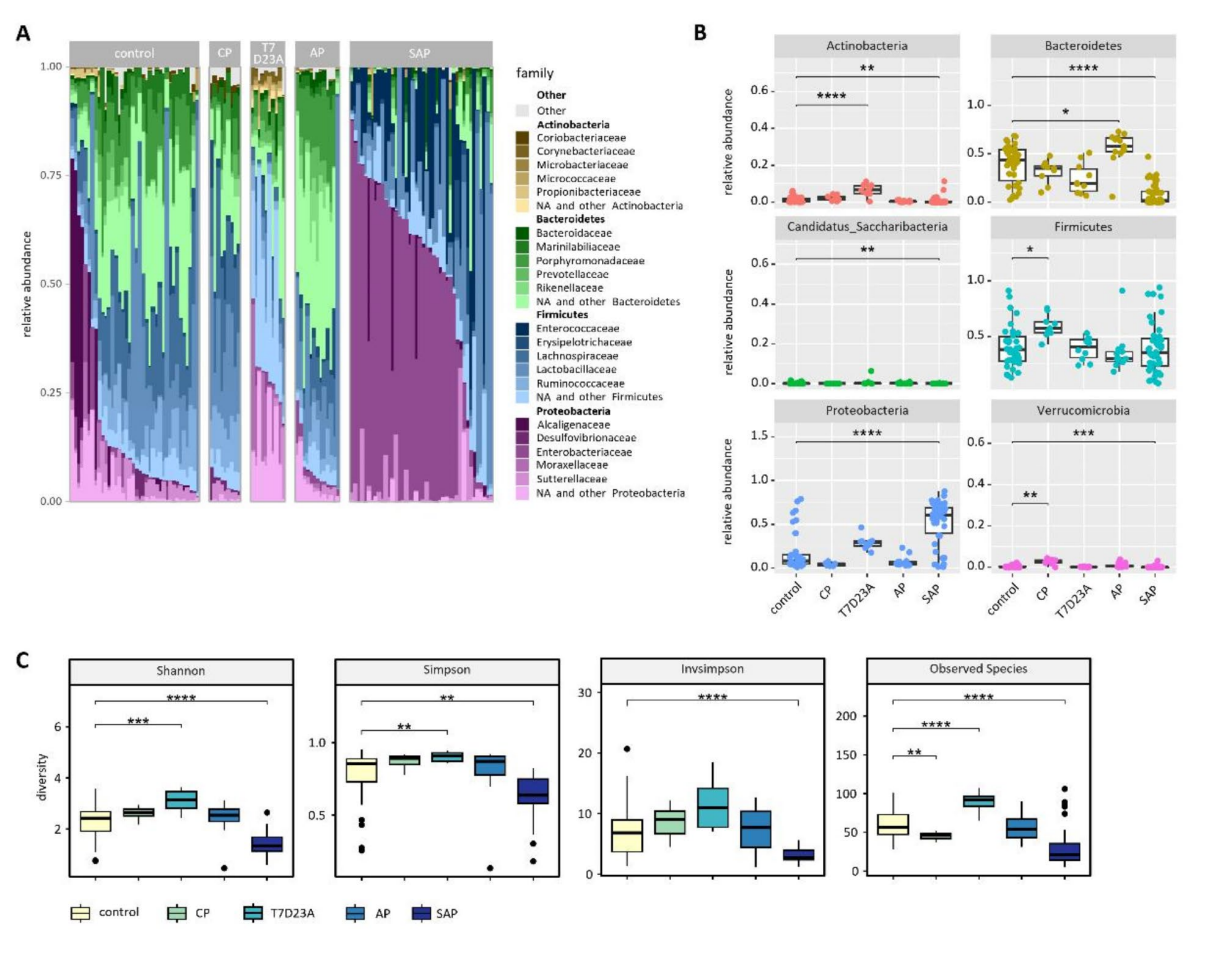
Changes of the duodenal microbiome in experimental pancreatitis models. (**A**) A stacked bar graph shows the most abundant taxa of the four most prominent phyla. (**B**) Box plots illustrate the relative abundance of the six most prominent phyla in general. (**C**) Box plots show the diversity index (Shannon, Simpson and Invsimpson) and the richness (observed species) in duodenum samples. Significant changes were tested by Wilcoxon-test followed by correction for multiple testing.

In contrast to the colon samples, we observed substantial microbiome changes in the duodenum between the different pancreatitis models. SAP mice as well as the T7D23A animals showed significant differences compared to controls. Stacked Bar Plot illustrates the top 4 Phyla with the 5 most represented families of duodenal samples (Fig. 4A). A Box plot illustrates the observed differences of the 6 most common phyla between acute and chronic pancreatitis (Fig. 4B). Unlike the colon samples, the duodenum samples revealed differences with respect to diversity and richness (Fig. 4C). In particular, the duodenum of SAP animals showed significantly reduced diversity, while the diversity in T7D23A animals seemed rather increased.

## Impact of pancreatic function and acute immune response on the microbiome

**Fig. 5:**
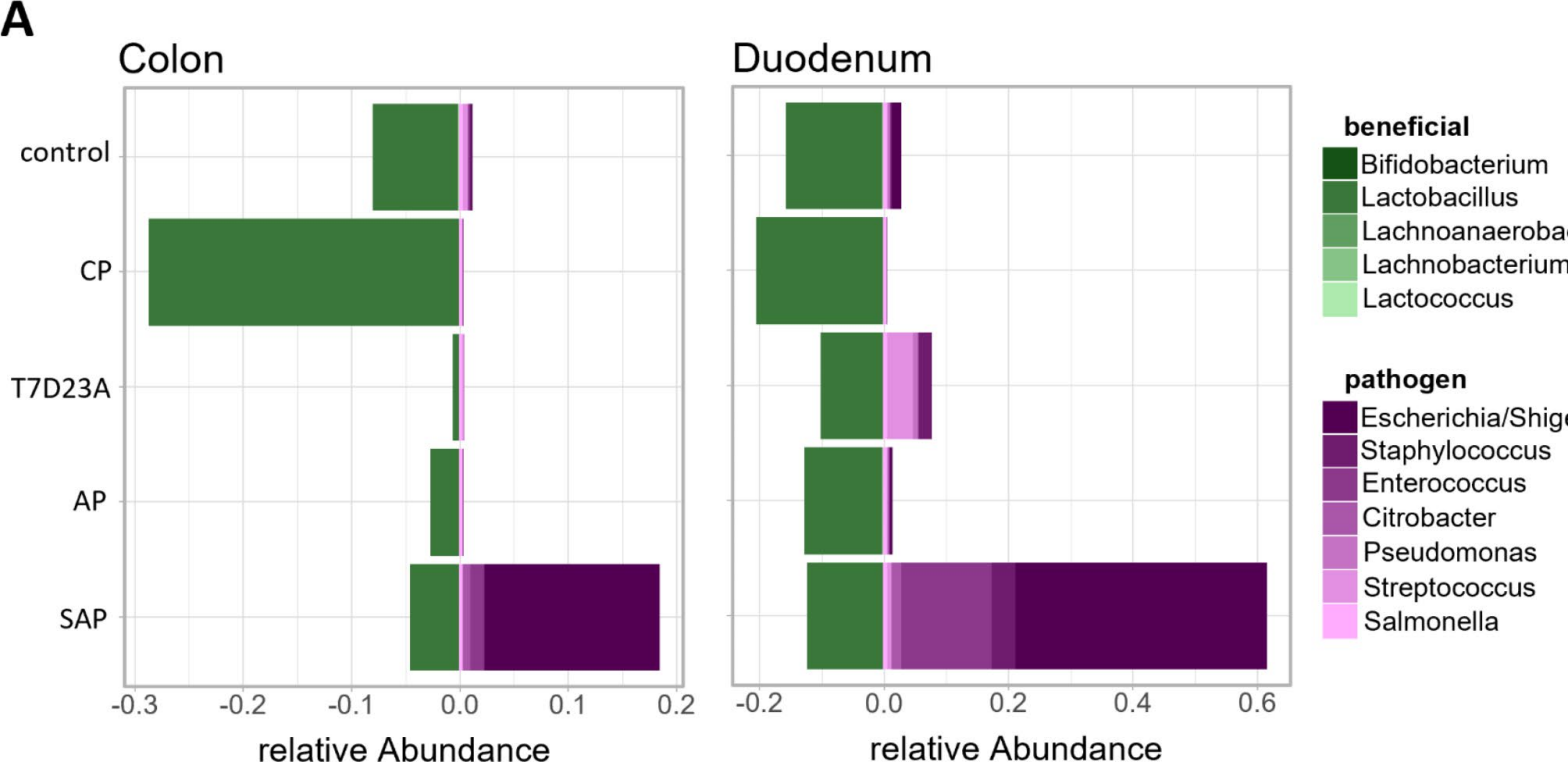
Gut microbiome changes in mouse models of acute and chronic pancreatitis. (**A**) A stacked bar graph illustrates the relative abundances of beneficial (green) and facultative pathogen taxa (purple) in all used models of AP and CP as well as controls.

The most significant changes in the gut microbiome were observed in colonic and duodenal samples of severe acute pancreatitis (SAP) animals. In the SAP model we observed mainly, an increase of facultative pathogenic bacteria (*Escherichia/Shigella*, *Staphylococcus*, *Enterococcus*, *Citrobacter*, *Pseudomonas*, *Streptococcus* and *Salmonella*) (Fig. 5A). The relative abundance of beneficial taxa such as *Bifidobacterium*, *Lactobacillus*, *Lachnoanaerobaculum*, *Lachnobacterium* and *Lactococcus*, on the other hand, was reduced in all pancreatitis models except in the colon samples of caerulein-induced CP animals (Fig. 5A).

## Discussion

It is known that diseases of the gastrointestinal tract such as pancreatitis are associated with changes in the microbiome^7,9,19^. In this study, we compared acute and chronic disease models of pancreatitis to investigate the influence of pancreatic function and disease severity on the gut microbiome. We compared the composition of the gut microbiome in the colon and duodenal part of the gut. Our results suggest that the general dysbiosis during mild acute and chronic pancreatitis is related to a pancreatic dysfunction, whereas the overgrowth of facultative pathogens in SAP is driven by the systemic immune response. In a previous study we could show that the composition of the intestinal microbiome is closely connected to the pancreatic secretory function^7^. Differences in pancreatic elastase levels associated with greater changes in microbiota diversity than with age, body mass index, sex, smoking, alcohol consumption, or dietary factors. The secretion of digestive enzymes, for which elastase is only a marker enzyme, determines at least in part the nutrition supply for the intestinal bacteria^8^. In addition to digestive enzymes, the pancreas secretes bicarbonate, which buffers the pH of the gastric juice^20^, and antimicrobial peptides such as CRAMP^21^, which both influence the intestinal microbiome. In our study we utilized different murine models of experimental acute or chronic pancreatitis to compare the influence of dysfunctional pancreatic secretion, and acute inflammatory responses on the microbial composition in the colon and duodenum.

Chronic pancreatitis is accompanied by a gradual loss of functional pancreatic tissue which ultimately leads to exocrine insufficiency. A declining pancreatic exocrine function results in changes of the intestinal microbiome, as the lack of digestive enzyme secretion alters the substrate for intestinal bacterial growth^8^. CP-mice induced via repetitive caerulein injections show a strong reduction of functional exocrine tissue, but not a complete loss of function^18,22^. In contrast to the caerulein model, transgenic T7D23A mice have a pronounced loss of exocrine function which reflects the late stage of CP^17^. In histologic sections, almost no remaining exocrine tissue could be observed^17^. Both models of CP show changes in the intestinal microbial composition, which is in line with findings of dysbiosis and increased abundance of facultative pathogenic strains, associated with a changed metabolic pattern, in patients with chronic pancreatitis^9,23^. Transgenic T7D23A animals showed an increase in facultative pathogenic strains in the duodenum, which we could not observe in caerulein induced CP mice. One possible explanation is the almost complete loss of the exocrine secretory tissue in T7D23A animals, which in addition to the lack of digestive enzymes, also have a greatly reduced secretion of antimicrobial peptides from acinar cells^21^. This may explain the greater impact on the gut microbiome composition. The CP models prove that the pancreatic function has a direct impact on the composition of the gut microbiome. In T7D23A transgenic mice, the absence of an inflammatory response allowed us to evaluate the direct effect of pancreatic function on the gut microbiome. Ritz et al. demonstrated in a porcine model that a dramatic shift in the microbiome composition due to pancreatic insufficiency could be reversed by enzyme replacement^8^. In contrast to the porcine model, we did not observe such marked changes in microbial diversity, which may be due to the fact that rodents are coprophagous, allowing recurrent colonisation of the intestinal microbiome.

Whereas the permanent loss of pancreatic function is a hallmark of CP, AP is characterized by a temporary impairment of pancreatic secretion and a prominent systemic immune response^10,13,14^. Interestingly we also observed in the mouse models of AP severity dependent changes of the gut microbiome composition. The most significant effect could be observed in the SAP model, which is accompanied by a pronounced systemic immune response^13,24,25^. A strong reduction of intestinal immune cells being the consequence of an induced immune suppression, allows bacteria to translocate the intestinal barrier and colonize pancreatic necrosis^10^. In the partial duct ligation model a residual secretion of pancreatic enzymes remains, so that the observed microbiome changes are presumably caused by the immune response rather than by a pancreatic dysfunction. An increase of facultative pathogens especially of proteobacteria could be detected in these mice, similar to other severe inflammatory reactions such as sepsis^26^, severe trauma^27^ or burn wounds^28^. All of these clinical phenotypes are accompanied by a prominent inflammatory response like the one we observed in SAP^10,13^. In a mild form of AP we detected only minor alteration in the gut microbiome, comparable to the changes seen in the model of CP, which makes it likely that the short-term secretion blockade is responsible for these changes. The absence of facultative pathogens suggests a functional immune response in the intestine in contrast to the SAP-model. Other studies have shown that general disease-related changes in the gut microbiome are not necessarily disease-specific^29^. Apparently, it`s the strength of the systemic immune response which has an impact on the composition of the intestinal microbiome. An increase of facultative pathogens like *Staphylococcus* can be observed in patients with severe SIRS^30^. A systemic immune response or SIRS are not specific to AP and can also be observed in severe trauma or sepsis. These results suggest a significant impact of the immune response on the gut microbiome in a severity dependent manner. This effect of the systemic immune response is significantly more pronounced than the influence of pancreatic secretory function on the composition of the gut microbiome and is most evident in the duodenum, which is localized directly beside the inflamed pancreas.

Apparently, the influence of pancreatic function and the inflammatory response on the gut microbiome has a bidirectional aspect, as the microbiome can also have an impact on the immune response, which has been shown for pancreatic cancer^31,32^. Microbiota derived metabolites modulate the immune response^33^ and can therefore influence the disease course and outcome. Notably, short chain fatty acids (SCFAs) have been shown to act on various immune cells of the innate and the adaptive immune system and influence the local and systemic immune response which could affect severity and outcome of acute and chronic pancreatitis^10,13,14,16,18^. Currently, we are just beginning to understand how microbiome and immune response interact. In the duodenum of mice experiencing SAP we observed a significant decrease of taxa which are known to produce SCFAs and this change of metabolites could trigger the systemic immune response. In addition, we observed a significantly increased abundance of *Escherichia/Shigella*, a known producer of Trimethylamin-N-oxide (TMAO)^34,35^, and another microbiota-derived metabolite from dietary phosphatidylcholine, which is particularly abundant in red meat and egg yolks, and has been identified as a predictor of cardiovascular diseases^36^. Evidence points to a NLRP3 inflammasome activation leading to endothelial dysfunction^37^. Other diseases associated with TMAO levels include diabetes mellitus and chronic kidney disease^38^. Branched-chain amino acids (BCAAs) coming from both dietary consumption and gut microorganism biosynthesis. Facultative pathogens such as *Staphylococcus*, *Streptococcus*, *Escherichia/Shigella*, *Klebsiella* or *Selenomonas* are main producers of BCAAs^39^ and were significantly increased in SAP. BCAAs act as nutrition sensor and can regulate via mTOR signalling^40^, cell proliferation, survival, protein synthesis and autophagy, which plays an important role for disease outcome^41,42^.

Our study has several limitations due to the fact that the murine microbiome is not generally comparable to the human microbiome. Mice are coprophagous animals that redigest their own faeces and recolonise their microbiome, so microbiome transfer via co-housing is possible in rodents^43^. For this reason, the microbiome of mice is significantly different from humans, especially in the upper intestinal tract. Furthermore, the food spectrum of mice is different from that of humans. However, comparative studies have shown that the murine and human microbiomes are comparable, with about 90% similarity in detectable phyla and genera^44^. In general, a greater Bacteroidetes/Firmicutes ratio is seen in humans which is reversed in mice^45^. However, advantages of murine studies are the homogeneous genetic background of the animals and the standardisation of the environmental conditions such as diet composition and equal treatment of all animals within the group. A further limitation of our study is the utilization of disease models with different ways of inducing pancreatitis. While i.p. injections are used in the case of caerulein-induced mild pancreatitis, surgical interventions are needed for the induction of SAP. So far, no standardised animal model with a variable pancreatitis severity could be established, therefore different models are needed to investigate severity differences in animals. Another point that animal studies are not able to depict are the various clinical factors that influence the microbiome, such as treatments with antibiotics, pain medication, enteral nutrition, treatment with proton pump inhibitors or endoscopic interventions. However, the aim of this study was not to investigate the influence of these therapeutic parameters on the microbiome, but to investigate the impact of pancreatitis-associated factors. Animal models have a lower bias and variation compared to human studies but on the other hand cannot reflect all possible influences of clinical treatments.

In conclusion, our data suggests that pancreatic function has a direct influence on the composition of the gut microbiome. The development of pancreatic insufficiency during CP is associated with significant changes in the microbiome. In addition, acute inflammatory responses affect the microbial composition in the gut in a severity dependent manner. Especially in severe episodes of AP facultative pathogens like *Enterococcus* or *Escherichia/Shigella* show increased abundance compared to controls. Future studies need to clarify to what extent a dysbiotic gut microbiome can be used as a diagnostic tool for pancreatic function during disease progression of CP as well as a marker for disease severity in cases of AP or acute episodes of CP.

## Materials and methods

### Ethics declaration

All animal experiments were approved by the local animal care committee (Landesamt für Landwirtschaft, Lebensmittelsicherheit und Fischerei Mecklenburg-Vorpommern LALLF-7221.3-1-011/17, LALLF-7221.3-1-014/19, LALLF-7221.3-1-008/21, LALLF-7221.3-1-030/22) and performed in accordance with the national guidelines for animal experiments and the ARRIVE guidelines.

### Animal model

C57BL/6-J mice were obtained from Charles River (Sulzfeld, Germany). All mice were maintained under pathogen-free conditions in ventilated animal cabinets with a 12-h light-dark cycle at a temperature of 21–24 °C (humidity 50–70%) and with access to food and water ad libitum. 8–12 weeks old male mice were used for the experiments. All mice were given a pain medication consisting of tramadol (1 mg/ml) in their drinking water. The control group contains untreated mice as well as sham operated mice without induction of SAP.

## Acute pancreatitis

AP was induced by hourly intraperitoneal injections of caerulein (4030451, Bachem) (50 µg/kg/bodyweight) to a maximum of 8 h for three consecutive days. AP animals were sacrificed 72 h after the first caerulein injection, as previously described^10^.

SAP was induced by partial pancreatic duct ligation followed by a single injection of caerulein (50 µg/kg/bodyweight) 48 h after surgery, as previously described^10,13,24,46^. The animals were anesthetized with ketamine/xylazine, the peritoneal cavity was surgically opened, and the pancreas was exposed. The pancreatic duct was ligated at the junction between the gastric and the duodenal lobe as previously described^46^. 48 h after surgery the animals received a single i.p. injection of caerulein (50 µg/kg/body weight). 72 h after the duct ligation the mice were sacrificed.

## Chronic pancreatitis

CP was induced by 6 hourly repetitive injections of caerulein (50 µg/kg/body weight), three days a week over a period of 4 weeks, as previously described^18^. Animals were sacrificed four weeks (28d) after the first caerulein injection.

As a second model of CP we used T7D23A heterozygote mice, which show a nearly complete replacement of exocrine pancreatic tissue by fatty tissue at the age of 12 weeks^17^. All mice were sacrificed at an age of 12–14 weeks.

### Sample preparation

PSP Spin Stool DNA kit from Stratec Molecular GmbH (Berlin, Germany) was used for the isolation of DNA from murine duodenal and colonic samples. Stool DNA was used for amplification of the V1-V2 region of bacterial 16 S rRNA genes and sequenced on a MiSeq platform (Illumina, San Diego, USA) as previously described^7,10,19^. Sequencing data were processed using the DADA2 package (v.1.14) for R (v. 3.61)^47^. Reverse reads were trimmed to 230 and 180 bp, respectively, or at the first base with a quality of 5. Reads-pairs with ambiguous base calls or expected errors larger than 2 were discarded. Error profiles were inferred from 10⁹ bases from randomly selected clean forward and reverse reads separately, which were subsequently used for read correction and inference of amplicon sequence variants (ASV). A de novo consensus-based approach was used to identified chimeric reads and removed them from the dataset to create the final ASV-by-sample count table. Taxonomic annotations of ASV sequences were inferred using the Bayesian classifier of the DADA2 package and the Ribosomal Database Project (RDP) release 16 database^48^. Only samples with more than 5.000 clean reads were included in our further analysis. ASV were summarized on genus level for further analysis, the data have been attached to the manuscript as supplementary file 1.

### Statistics

All statistical analyses were performed with R version 4.2.3 in RStudio and all figures were visualized with the packages “ggplot2” (version 3.4.4)^49^, “fantaxtic” (version 2.0.1)^50^ and „graphics”. The top 20 taxa with the highest fold change in relative abundance in CP or AP in relation to the control were calculated using permutative multivariate analysis of variance (PERMANOVA, permutations = 1000, distance = “bray”), which was implemented using the “adonis” function in the R package “vegan” (version 2.6-4)^51^. This R package was also used to calculate the alpha diversity with the diversity indices ‘Shannon’, ‘Simpson’ and ‘Inverse Simpson’ as well as the richness of the microbiome with the functions “diversity” and “specnumber” and examined with the two-sample unpaired t-test (R package “rstatix” version 0.7.2.999) to the control. The p-value was adjusted according to the ‚Holm‘ method. The functions „decostand”, “vegdist” (method = „bray“) of the „vegan“ package and „cmdscale” in the „stats“ package were used for the Principal Coordinates Analysis (PCoA). To visualize the changes in the microbiome in the experimental pancreatitis models, a phyloseq file was created from the original file using the R package “phyloseq” (version 1.42.0)^52^ and „ape“ in the R package „ape“ (Version 5.7-1)^53^. The relative abundance of the six most abundant phyla and the relative abundances of beneficial and facultative pathogenic taxa were tested for significance using the two-sampled unpaired Wilcox test, implemented by the function “wilcox_test” in the “rstatix” packages, compared to the control. The results of the statistical tests with adjusted p < 0.05 according to ‘Holm’ were considered significant. All statistical analyses were performed using R-studio. Calculation of ‘Shannon diversity’ and ‘Richness’ as well as the PCoA (Bray-Curtis or Jaccard) were performed by using the R package ‘vegan’.

### Histology staining procedure

Azan blue and H&E staining’s were done on 2 μm microtome sections from paraffin embedded pancreatic tissue samples. Tissue sections were scanned with Pannoramic MIDI II (Sysmex) slidescanner.

## Electronic supplementary material

Below is the link to the electronic supplementary material.

Supplementary Material 1

## Data Availability

All data generated or analysed during this study are included in this published article and the supplementary files.
